# Sovereign AI supercomputers: a global landscape review of unprecedented biomedical research infrastructure

**DOI:** 10.3389/frai.2026.1796682

**Published:** 2026-06-03

**Authors:** Lansaol Yang, Michael E. Bryan, Eduardo Veiga, Ian Lowenhoff, Alex Wan, Isam Mina, Tracey Allen, Javier Antonio Alfaro, Gareth Bloomfield, Julian Beach, Kristen Dahlgren, Nick K. Davis, Elisa Fontana, Spyridon Gennatas, Qamar Ghafoor, Franck Housseau, Daniel Lubelski, Zhehao Zhang, Matt Hancock, William Ince, Dominic James, Sam Khan, Victoria Kunene, John McGrane, Gerard Cathal Millen, Benjamin Moxley-Wyles, David Narganes-Carlon, Miranda Payne, Paul J. Ross, Rene Roux, Michael Rowe, Rebecca Lee, Jerry S. H. Lee, Justin K. H. Liu, Deepak Aggarwal, Aaron B. S. Teoh, Chrissie Thirlwell, Michael Tilby, Stefan Symeonides, Isabella Watts, David B. Agus, Santa J. Ono, Tim Elliott, Paul Calleja, Lennard Y. W. Lee

**Affiliations:** 1 Department of Neurosurgery, Johns Hopkins University, Baltimore, MD, United States; 2 Centre for Immuno-Oncology, University of Oxford, Oxford, United Kingdom; 3 Ellison Institute of Technology, Oxford, United Kingdom; 4 Broad Institute of MIT and Harvard, Cambridge, MA, United States; 5 Research Computing Services, University of Cambridge, Cambridge, United Kingdom; 6 Rhodes House, University of Oxford, Oxford, United Kingdom; 7 Houses of Westminster, London, United Kingdom; 8 Department of Biochemistry and Molecular Biology, Cumming School of Medicine, University of Calgary, Calgary, AB, Canada; 9 Medicines and Healthcare Products Regulatory Agency, London, United Kingdom; 10 Cancer Vaccine Coalition, Middlebury, VT, United States; 11 Department of Biological Engineering, Massachusetts Institute of Technology, Cambridge, MA, United States; 12 Sarah Cannon Research Institute, London, United Kingdom; 13 Guy's and St Thomas' NHS Foundation Trust, London, United Kingdom; 14 Department of Oncology, University Hospitals Birmingham NHS Foundation Trust, Birmingham, United Kingdom; 15 Laboratory of Cell Physiology, ONCOLille Institute, University of Lille, Lille, France; 16 Department of Oncology, Johns Hopkins University, Baltimore, MD, United States; 17 Havilland Health, London, United Kingdom; 18 Oncology Centre, Addenbrooke's Hospital, Cambridge University Hospitals NHS Foundation Trust, Cambridge, United Kingdom; 19 Health in Hyperdrive, London, United Kingdom; 20 Leicester Cancer Research Centre, University of Leicester, Leicester, United Kingdom; 21 Department of Cancer and Genomic Sciences, University of Birmingham, Birmingham, United Kingdom; 22 Royal Cornwall Hospitals NHS Trust, Truro, United Kingdom; 23 Department of Paediatric Oncology, Birmingham Children’s Hospital, Birmingham, United Kingdom; 24 Department of Cellular Pathology, Oxford University Hospitals, Oxford, United Kingdom; 25 Department of Oncology, Churchill Hospital, Oxford University Hospitals, Oxford, United Kingdom; 26 Oxford Cancer and Haematology Centre, Oxford University Hospitals NHS Foundation Trust, Oxford, United Kingdom; 27 The Christie NHS Foundation Trust, Manchester, United Kingdom; 28 Ellison Medical Institute, Los Angeles, CA, United States; 29 Keck School of Medicine, University of Southern California, Los Angeles, CA, United States; 30 Clinical Effectiveness Unit (CEU), National Cancer Audit Collaborating Centre (NATCAN), Royal College of Surgeons of England, London, United Kingdom; 31 The Clatterbridge Cancer Centre, Liverpool, United Kingdom; 32 Bristol Medical School, University of Bristol, Bristol, United Kingdom; 33 Arden Cancer Centre, University Hospitals Coventry and Warwickshire, Coventry, United Kingdom; 34 Cancer Research UK Scotland Centre, Institute of Genetics and Cancer, University of Edinburgh, Edinburgh, United Kingdom; 35 Department of Oncology, Imperial College Healthcare NHS Trust, London, United Kingdom

**Keywords:** artificial intelligence for science, bioinformatics, biomedical big data, drug discovery, high-performance computing, precision medicine, sovereign AI supercomputers, vaccine development

## Abstract

Artificial intelligence (AI) has rapidly become the focal point of global governmental attention and investment. Nations are launching AI for science strategies on a scale comparable to historic endeavors such as Apollo and the Manhattan Project. These coordinated programs carry profound promise for people living with cancer, for those at risk of disease and for transformative public benefit. Central to this transformation is the rise of sovereign AI supercomputers which are fundamentally reshaping biomedical research. These publicly owned systems provide secure, large-scale computational capacity, enabling integration of complex health data and rapid analysis that was previously constrained. This review examines the geographic distribution, technical capabilities, and biomedical applications of these infrastructures. Key computational workloads that now benefit significantly from AI implementations include cancer imaging and diagnosis, personalized treatments, whole-genome and single-cell level analysis, and computational drug discovery. This approach has supercharged our efforts at the United Kingdom’s Cancer Vaccine AI & Supercomputing Project, our flagship national initiative to create new AI foundation models to accelerate the development of tools to establish immunity from cancer. In addition, this review evaluates governance models that safeguard patient privacy and intellectual property as well as measures that promote international collaboration while preserving compliance with regional regulations and make safer, more precise and effective treatments for public benefit. Substantial challenges exist, however, including inequitable resource availability, heterogeneous data standards and regulatory frameworks, and unbalanced computational expertise impeding the effective use of sovereign compute. Global collaborations are key to providing equitable access to advanced analytics, shortening the path from bench to bedside, and developing critical innovative tools for people affected by cancer.

## 1 Introduction

Artificial intelligence (AI) has become a central driver of global scientific progress, with national priorities, strategies and investment all orienting it as the foundation of a new research era. Large scale initiatives, such as the United States (US)’s Genesis Mission and the United Kingdom (UK)’s AI for Science Strategy, exemplify this movement and reflect the growing ambition in advanced infrastructure to unlock new capabilities, particularly in health and cancer research (The White House, 2025; Department for Science IT, 2025). This momentum has elevated sovereign AI supercomputers to the status of strategic scientific assets. These sovereign high-performance computing (HPC) systems represent a distinct category of computational infrastructure that is publicly owned, nationally funded, and operated under a country’s jurisdiction, serving the priorities of the host nations (Li and Yang, 2021). They are subject to domestic regulations administered by national institutions, which ensure a high degree of autonomy, safeguard sensitive data within national legal frameworks, and promote technological independence (Li and Yang, 2021). Their placement reflects national research priorities, proximity to innovation centers, and levels of investment.

**Figure 1:**
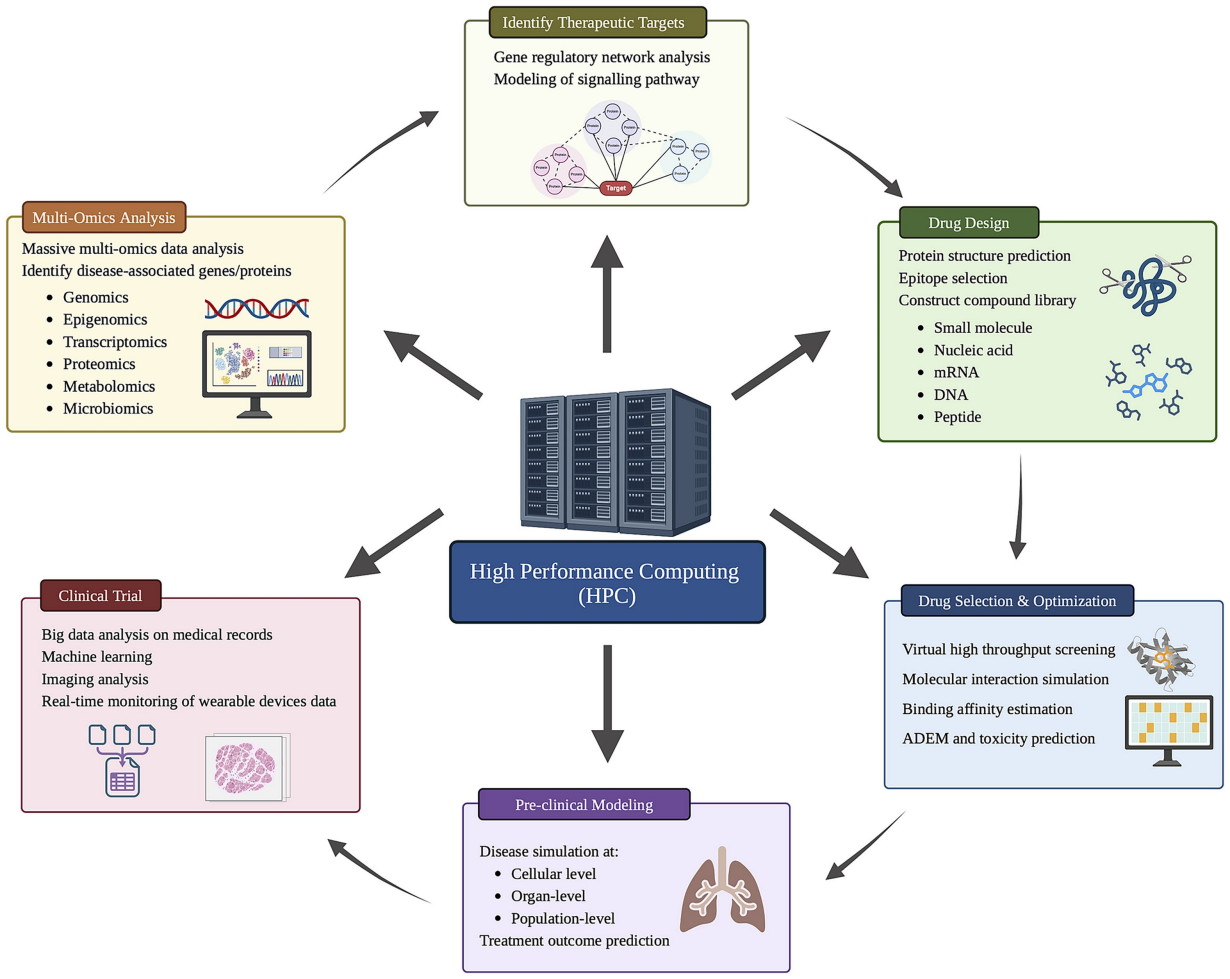
Schematic overview of the integration of HPC across the modern drug discovery pipeline. Created by Biorender with the license uploaded in the discussion forum (Citation: Created in BioRender. Yang, L. (2026) https://BioRender.com/2xafjpl.

Supercomputers now support transformative applications that involves intensive data processing and complex modeling, including climate science, astrophysics, nuclear research, materials science, and biomedical research (Jonathan Carter et al., 2025; Melo and Bernardi, 2023). Specifically, they offer secure environments for analyzing sensitive health datasets and AI-driven tasks such as training complex models, constructing digital twins, molecular simulations, and rapid drug design, bringing far-reaching impact on cancer research and biomedical innovation (Figure 1) (Ares de Parga et al., 2025; Liu et al., 2016). For instance, during one of the most significant public health crises of this century, the SARS-CoV-2 outbreak, the Summit system transformed pandemic response and antiviral drug discovery to an unprecedented speed by enabling rapid simulation of viral spike protein and rapid virtual screening of billions of compounds within days, compressing timelines that historically required months or years (Melo and Bernardi, 2023; Acharya et al., 2020). Within this landscape, the success of anti-SARS-CoV-2 vaccines greatly boosted public interest in mRNA vaccine development. Two recent studies demonstrate that AI driven pipelines not just accelerate, but also streamline the full mRNA vaccine development workflow, from selection and prediction of antigen and epitope to design and optimization of mRNA construct, immunogenic adjuvant and delivery vehicles, and downstream safety and efficacy modeling (Imani et al., 2025; Olawade et al., 2024). Multiple initiatives have been moving forward, for instance, Aurora is being directed toward the development of broad-spectrum antiviral vaccines in partnership with University of Chicago, and Dawn from UK is extending the integration of HPC and AI into mRNA vaccine research beyond infectious disease to oncology, dedicating to the development of personalized cancer vaccine (Argonne National Laboratory, 2026; University of Oxford, 2025). On the other hand, recent breakthroughs such as AlphaFold and its successor AlphaFold3, together with emerging AlphaGenome, exemplify how AI enabled supercomputing is fundamentally redefining structural biology. While earlier approaches were limited by experimental constraints and low throughput, AlphaFold3 extends accurate structure prediction beyond proteins to protein complexes, nucleic acids, and ligand interactions. This effectively moves the field from static structure determination toward modeling functional molecular assemblies, which are the true units of biological activity (Krokidis et al., 2025). In parallel, AlphaGenome extends this paradigm to the genomic scale by predicting thousands of functional outputs directly from DNA sequence, including gene expression, chromatin accessibility, transcription factor binding, and splicing, at single basepair resolution (Avsec et al., 2026). By linking sequence variation to molecular function across regulatory and non-coding regions, it addresses a long-standing gap between genotype and molecular phenotype. Together, this synergy of HPC and AI signals a new era in move biomedical science from observation to prediction, and from discovery to delivery, with profound implications for human health worldwide.

**Figure 2:**
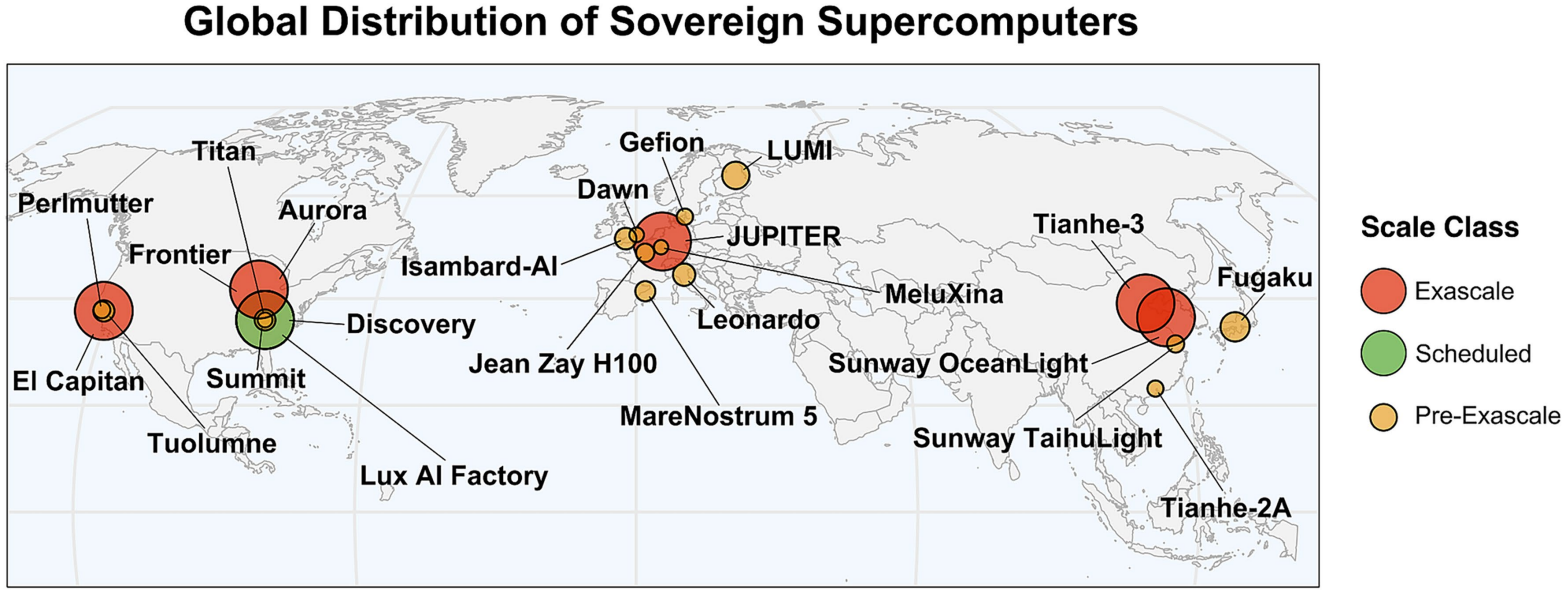
Distribution of major sovereign supercomputing systems supporting biomedical science across North America, Europe, and Asia. Each bubble is positioned at the geographic location of a sovereign supercomputer. Red indicates operating exascale-class systems, green indicates systems under development, and yellow indicates operating pre-exascale systems. Bubble diameter corresponds to peak performance. Created by R studio by authors.

AI Supercomputers have laid the foundations for the establishment of major national initiatives, like the UK Cancer Vaccine AI & Supercomputing Project. This project is creating new foundation models to accelerate the development of tools to pioneer immunity from cancer and make safer, more precise, and effective treatments for public benefit. Using the Dawn AI Supercomputer, it has trained models using 120 million peptide–HLA pairs from over 12,000 tumors through an interdisciplinary consortium of 1,500 scientists, industry, government clinicians, patients, and charity leads (University of Oxford, 2025). Despite substantial progress, supercomputers remain unevenly distributed worldwide (Figure 2). Such imbalance can underrepresent certain ethnic groups, overlook regional differences in disease patterns, and fail to reflect changing population characteristics over time, thereby biasing model baselines. To elaborate on this issue, this review examines major sovereign supercomputers supporting biomedical research (Table 1). By analyzing their global distribution, we aim to assess preparedness for shared health challenges, identify geographic disparities in computational capacity, and inform policies that promote equitable and effective research infrastructure.

**Table 1:** Global sovereign supercomputing systems and their biomedical research applications.

Supercomputer name	Country	Organization	Rmax (petaFLOPS)	Biomedical research highlights
Perlmutter	US	NERSC	79	Support structural biology to reveal conformational changes in the viral replication transcription complex and accelerate cryo-EM analysis during SARS-CoV-2, facilitating antiviral drug development.
Titan	US	ORNL	27	Molecular dynamics simulation, facilitated the identification of a key cancer-related signaling protein, c-Src kinase.
Summit	US	ORNL	200	AI-integrated simulation, training of machine learning models to and supporting systems biology studies that identify clinical and genomic factors associating with cancer, Alzheimer’s, heart disease, and addiction.
Frontier	US	ORNL	1,353	Part of the DOE-NCI CANDLE initiative, supports AI-driven graph analytics for PubMed/SPOKE, trains deep learning models for clinical records to uncover symptom–treatment–outcome associations, empowers genome-wide epistasis studies to identify pathogenic genetic variants, advances precision medicine.
Aurora	US	Argonne National Lab	1,012	Part of the CANDLE and IDEAL initiatives, AI-driven design of therapeutic small molecules, supports HARVEY model for cancer metastasis study.
Tuolumne	US	LLNL	208	Dedicated to open science, enabling cancer drug discovery, computational biology, and biosciences through AI-driven molecular screening, protein modeling, and OpenFold3-based training for advanced protein folding simulation.
El Capitan	US	LLNL	1,809	High-throughput structural biology and therapeutic discovery. Empower ElMerFold for large-scale protein structure prediction using OpenFold3 and support GUIDE platform in discovering small molecules and antibody-based therapeutics.
Discovery	US	ORNL	TBD	Multi-exaflop AI & simulation, protein structure prediction, synthetic biology.
Lux	US	ORNL	TBD	AI model training for precision medicine, generative therapeutics.
Jean Zay H100	France	IDRIS	125·9	Strategic AI development across domains including biology and physics, and biomedical applications such as large-scale natural language processing, image processing, and protein interaction modeling during SARS-CoV-2 pandemic.
LUMI	Finland	EuroHPC JU / CSC	386	Advances onco-histopathology image analysis and diagnostic AI by powering projects ComPatAI and PanEncoder, and supports the development of a multimodal medical foundation model aimed at improving diagnostic accuracy and patient stratification in cancer care.
Leonardo	Italy	EuroHPC JU / CINECA Bologna	241	Partners with Dompé farmaceutici’s Exscalate platform in ultra-fast virtual screening, pandemic modeling, and molecular simulations; supports high-throughput design and testing of synthetic molecules for antiviral and oncology research, notably contributing to Exscalate4CoV, which advanced raloxifene into clinical trials.
Isambard-AI	UK	University of Bristol	217	Participate in the UK’s AIRR initiative, Isambard-AI delivers advanced infrastructure for academic and industrial innovation in AI-driven life sciences. Flagship projects include Nightingale AI at Imperial College, building a sovereign multimodal health model from NHS data; a scalable MRI-based prostate cancer screening initiative at UCL; and EgoAI at the University of Bristol, which analyzes data from wearables to support daily living and cognitive care.
Dawn	UK	University of Cambridge	19	Supported by UKRI and part of the AIRR alongside Isambard-AI, enables large-scale biomedical research through AI-powered digital twin modeling for personalized therapies and treatment prediction, as well as medical imaging workflows that accelerate biomarker discovery, disease stratification, and therapeutic development. Empowering next-generation cancer vaccine development.
JUPITER	Germany	Jülich Supercomputing Centre	1,000	Enables ultra-large molecular dynamics simulations of the nuclear pore complex at atomic resolution for drug and etiology research, empower the FAITH project, an AI-driven fatigue biomarker tracking in cancer patients, and support a large-scale brain foundation model integrating imaging and cellular anatomy to study neurological disease.
MareNostrum 5	Spain	Barcelona Supercomputing Center	175	Molecular modeling, functional prediction of genetic variants, and molecularly guided personalized medicine.
MeluXina	Luxembourg	LuxProvide	11	Breast cancer genomics, novel antibiotic discovery, data-driven personalized medicine.
Gefion	Denmark	Technical University of Denmark	67	Support protein engineering, synthetic biology, and large-scale biomedical data analysis, participate in initiatives that improve women’s health.
Tianhe-2A	China	National Supercomputing Center, Guangzhou	61	Genomics, biomedical big data, molecular dynamics, drug screening.
Tianhe-3	China	NSC, Tianjin	Exascale	Anticipated exascale biomedical workloads (no public benchmarks yet).
Sunway TaihuLight	China	NSC, Wuxi	93	Enhance quality control for massive sequencing data, support deep learning-based histopathology imaging analysis in cancer, and molecular simulations for novel therapeutics.
Sunway OceanLight	China	Qingdao Supercomputing Center	Exascale	No public benchmarks yet.
Fugaku	Japan	RIKEN Center for Computational Science	442	Enabled aerosol dispersion simulations that informed public health policy during the SARS-CoV-2 pandemic, support high-throughput drug screening and brain circuitry modeling to study neurological mechanisms.
AIRAWAT	India	C-DAC, Pune	8.5	AIRAWAT and PARAM Siddhi AI together represent a unified HPC ecosystem designed to support data intensive AI workflows, supporting large scale biomedical research, such as medical imaging processing, natural language processing, robotics and big healthcare data analysis.
Falcon series	The United Arab Emirates	Technology Innovation Institute, Abu Dhabi	N/A	Intensive national AI strategy leveraging Falcon large language models and the Condor Galaxy supercomputing network to enable rapid training of domain specific and multilingual models for applications in genomics and healthcare AI tools.
Santos Dumont	Brazil	LNCC, petropolis	14.3	Supported large scale bioinformatics, genomics and SARS-CoV-2 research, alongside national efforts leveraging large health datasets and public private collaborations to advance clinical and translational research.

Rmax, actual measured maximum performance; NERSC, Lawrence Berkeley National Lab; ORNL, Oak Ridge National Lab; LLNL, Lawrence Livermore National Lab; IDRIS, Institut du Développement et des Ressources en Informatique Scientifique; EuroHPC JU, European High Performance Computing Joint Undertaking; GUIDE, Generative Unconstrained Intelligent Drug Engineering; AIRR, AI Research Resource; UKRI, UK Research and Innovation; FAITH, Fatigue Therapy – AI-supported Diagnosis and Therapy of Tumor-associated Fatigue Syndrome; NSC, National Supercomputing Center; C-DAC, Centre for Development of Advanced Computing; LNCC, National Laboratory for Scientific Computation.

## 2 Method

HPC systems were identified using the TOP500 database, a widely recognized global benchmark for supercomputing (TOP500, n.d.). Systems ranked within the top 20 in peak performance at least once between June 2020 and November 2025 were screened, followed by a literature review to identify those applied in biomedical or AI driven research. The final selection was further categorized by continental distribution to provide a global perspective. Materials were eligible for screening if published in English or if an English translation was available.

## 3 Global snapshot of HPC capacity by region

**Figure 3:**
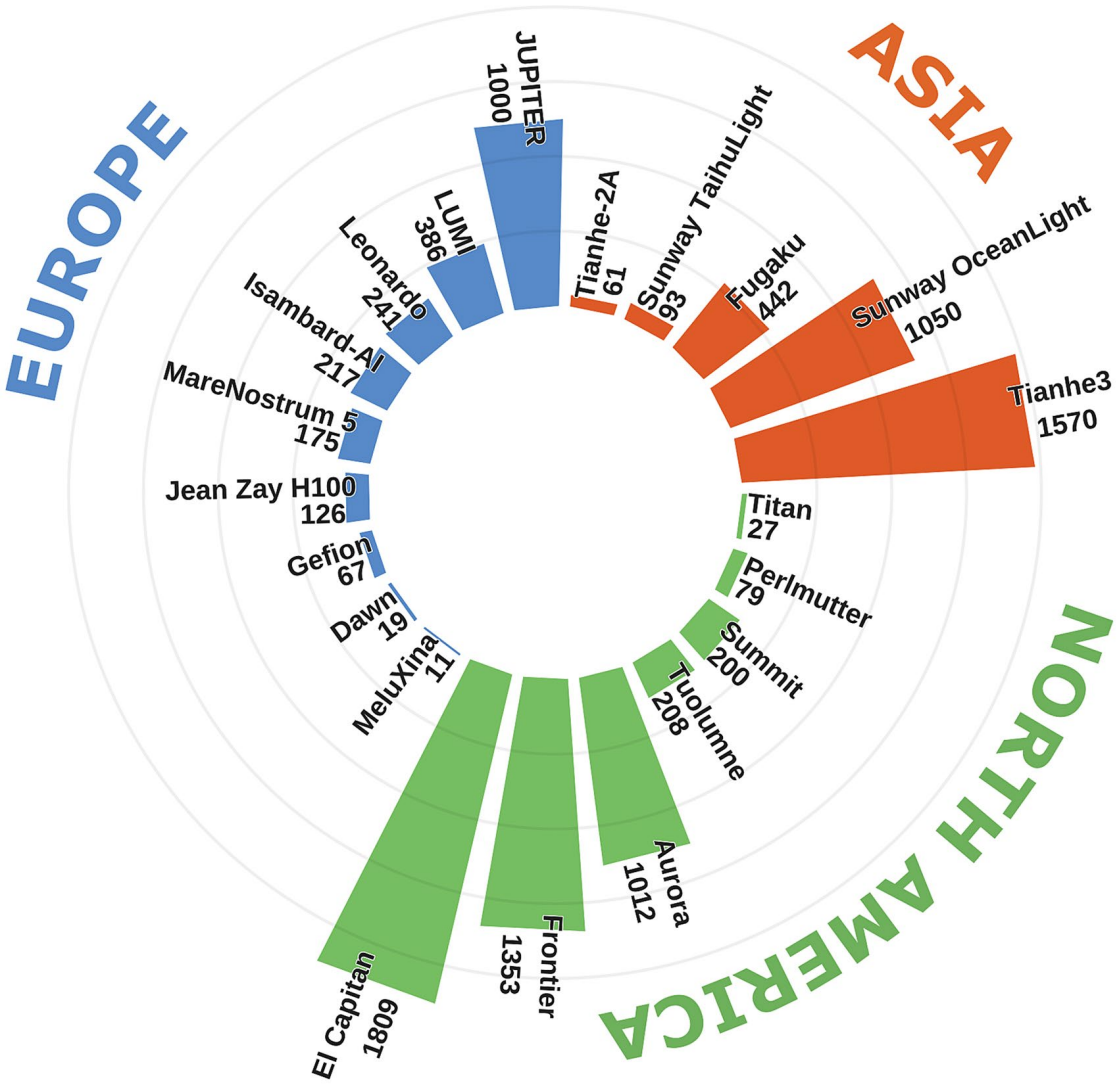
Illustration of major operating sovereign supercomputing systems supporting biomedical research and their computational capacities across continents. Bar length reflects the peak performance of each system, with numerical labels shown in petaFLOPS, while color denotes the continent of operation. Created by R studio by authors.

By 2025, global HPC capacity remains concentrated in North America, Europe, and Asia, accounting for 37.6, 32.3, and 27.2% of global capacity, respectively (Figure 3) (TOP500, n.d.). North America, led by the US, holds 35% of the system share and contributes nearly half (48.5%) of global computational power (TOP500, n.d.). This reflects a dense network of federally supported leadership systems in the US that closely link large scale biomedical research with structured national oversight. Europe has rapidly expanded its footprint by pursuing a distinct path that combines coordinated multinational initiatives such as the EuroHPC Joint Undertaking (EuroHPC JU) with a strong emphasis on sustainability in system design, making collaboration and sustainability core pillars of its sovereign computing strategy (EuroHPC, 2025). Asia represents another major contributor, driven largely by China and Japan, which host multiple top ranked supercomputers that support research in materials science, drug discovery, and climate modeling. On the other hand, regions such as South America, Africa, and Oceania host emerging HPC infrastructures that, although still limited in global rankings, are fostering important regional HPC initiatives that support growing biomedical research capacity (TOP500, n.d.). These disparities have direct consequences for biomedical research, influencing the pace of data driven discovery and shaping the extent to which scientists from underrepresented regions can participate in global research efforts.

### 3.1 North America

#### 3.1.1 US sovereign supercomputers for biomedical discovery

The US retains global leadership in sovereign supercomputing through a robust network of national laboratories and commercial platforms. During the early pre-exascale era, Perlmutter, a flagship system launched at the National Energy Research Scientific Computing Center (NERSC) in 2021, played a central role in structural biology by revealing conformational changes in the viral replication transcription complex and expediting cryo-electron microscopy data analysis during SARS-CoV-2 research, supporting antiviral drug development (Trifan et al., 2022). The upcoming system, NERSC 10 Doudna, named in honor of Jennifer Doudna’s pioneering contributions to CRISPR technology, is set to launch in 2026 with over tenfold the capacity of Perlmutter, advancing AI-enabled research (Ball, 2025).

The Oak Ridge National Laboratory (ORNL) has been at the forefront of HPC in the US, beginning with the launch of Jaguar supercomputer in 2005, which marked its entry into the peta-scale computing era (Jaguar, 2025). This was followed in 2012 by Titan, which is equipped with significantly enhanced GPUs and pioneering a hybrid CPU-GPU architecture to reach a peak performance exceeding 27 petaFLOPS (ORNL Launches Summit Supercomputer, n.d.; Turczyn, 2025). Titan made notable contributions to cancer research by revealing the first atomic-level ensemble of the intrinsically disordered N-terminal domain of c-Src kinase, a key cancer-related signaling protein (Turczyn, 2025). In 2018, the Summit system expanded these capabilities with approximately 200 petaFLOPS of peak performance and a unique architecture optimized for both simulation and AI workloads (ORNL Launches Summit Supercomputer, n.d.). This enabled Summit to support large scale machine learning-based discovery of cancer related factors and identification of genetic drivers of Alzheimer’s disease, cardiovascular disease, and addiction by integrating clinical and genomic data (ORNL Launches Summit Supercomputer, n.d.).

A pivotal milestone was research in 2022 with the commissioning of Frontier at ORNL, the world’s first confirmed exascale supercomputer with a performance of 1.35 exaFLOPS, marking a whole new computational era (TOP500, 2025a). As part of the Department of Energy (DOE) and National Cancer Institute’s Cancer Distributed Learning Environment (CANDLE) initiative, Frontier applied AI-driven graph analytics to massive datasets such as PubMed and SPOKE, uncovering previously unrecognized links between symptoms, treatments, and clinical outcomes (McDowell, 2025). It also powers genome-wide epistasis studies to identify disease-causing variants and trains deep learning models on millions of clinical records from cancer patients, accelerating progress in precision medicine (McDowell, 2025; Frontier, 2025).

In May 2024, Aurora at Argonne National Laboratory became the second confirmed exascale system, reaching a peak performance of approximately 1.012 exaFLOPS (Collins, 2025a). As part of the CANDLE initiative and the Integrated AI and Experimental Approaches for Targeting Intrinsically Disordered Proteins in Designing Anticancer Ligands (IDEAL) project, Aurora accelerates the development of novel cancer therapies by enabling AI-driven design and screening of small molecules for therapy-resistant proteins (Collins, 2025b). Aurora also powers the HARVEY model, a high resolution fluid dynamics platform developed at Duke University, which simulates blood flow and predicts metastatic seeding sites based on individual anatomical variation (Johnson, 2025). It advances early detection, personalized surveillance, and targeted intervention strategies for patients at risk of metastasis.

Lawrence Livermore National Laboratory (LLNL) hosts multiple cutting-edge supercomputers. Tuolumne and El Capitan, both deployed in 2024, represent a strategic expansion of computational capacity across unclassified and classified domains (Lawrence Livermore National Laboratory, 2025). Tuolumne is an unclassified counterpart of El Capitan, achieving approximately 208 petaFLOPS of peak performance (Lawrence Livermore National Laboratory, 2025). It supports a broad range of research in cancer drug discovery, molecular modeling, and AI-driven screening through protein folding simulation platforms such as OpenFold3 (Thomas, 2025). El Capitan, recognized in 2025 as the world’s fastest supercomputer, achieves a peak performance of 1.81 exaFLOPS (TOP500, 2025a). While primarily serving national security and nuclear stockpile stewardship, El Capitan also supports biomedical research such as powering the *ElMerFold* project that predict millions of protein structures at an exceptional speed using the OpenFold3 framework (Thomas, 2025). Beyond structural biology, both systems serve as foundational resources for LLNL’s bioscience and pharmaceutical discovery pipelines by supporting the Generative Unconstrained Intelligent Drug Engineering (GUIDE) platform, which integrates HPC and AI to accelerate small molecule and antibody-based therapeutic development (Ackerman et al., 2024).

Looking ahead, two next generation supercomputers, Lux AI Factory and Discovery, are being developed at ORNL through a strategic partnership between the DOE and industries to advance architectures optimized for AI-enabled research (Grabein and Stine, 2025). Lux, expected in 2026, will focus on training and refining large-scale AI models, while Discovery, planned for 2028, will deliver multi-exaflop performance for high throughput AI and simulation workloads (Grabein and Stine, 2025; Bethea, 2025). Both systems will support foundational language model training, generative AI for precision medicine, therapeutic compound design, large-scale chemical screening and protein structure prediction (Bethea, 2025; Valerie Taylor and Maccabe, 2019). Together, Lux and Discovery signify a transformative evolution in computational infrastructure, establishing AI not as a supplementary tool but as a core methodology in the scientific discovery process.

#### 3.1.2 Access and governance of sovereign supercomputers in the US

The governance of sovereign HPC infrastructure in the US is primarily orchestrated through the DOE, which oversees national user facilities and allocates access to leadership-class systems through competitive peer-review programs, which evaluate proposals based on scientific merit, scalability of applications, and broad national benefit (User Facilities, 2025; Ludwig, 2025). Central to this governance is alignment with federal data stewardship and ethical oversight frameworks established by the Federal Information Security Modernization Act (FISMA) of 2014 (Guo, 2024; U.S. Congress, 2014). Under this mandate, agencies must implement the National Institute of Standards and Technology (NIST) Risk Management Framework, which enforces standardized controls including identity authentication, audit logging, and incident response across HPC facilities (U.S. Congress, 2014). In 2024, the NIST issued Special Publication 800–223, introducing HPC-specific guidance that has been adopted by federal centers and commercial cloud providers to ensure confidentiality, integrity, and availability of federal data assets (Guo, 2024). To align with FISMA, the DOE operates an Unclassified Cybersecurity Program that applies NIST-based policies across its laboratories and supercomputing sites, promoting uniform cybersecurity standards, real-time monitoring, and risk mitigation (U.S. Department of Energy, 2024).

International use of US HPC systems is constrained by export control laws governing the export, reexport, and transfer of commodities, software, and technology to foreign entities (Bureau of Industry and Security, 2024a). The Export Control Reform Act of 2018 (ECRA) provides the legal basis for regulating dual use items under the Export Administration Regulations (EAR), under which entities on the Entity List generally require licenses for the export, reexport, or transfer of controlled technologies (Covington & Burling LLP, 2018; U.S. Congress, 2018; Bureau of Industry and Security, 2024b). In 2022, new restrictions imposed tighter controls on HPCs, graphics processors, advanced integrated circuits, and remote software delivery to designated foreign destinations (Bureau of Industry and Security, 2022). These measures reflect a strategic shift of the US toward tighter control over advanced computing technologies to safeguard national sovereignty and jurisdiction over sensitive resources.

### 3.2 Europe

#### 3.2.1 European sovereign supercomputers for biomedical research

Europe’s HPC ecosystem has expanded substantially, marked by the deployment of several flagship systems across key member states. These platforms not only enhance Europe’s digital autonomy but also support a growing array of biomedical research initiatives.

France was an early pioneer in sovereign HPC and launched Tera 100 in 2010, the nation’s first system to reach the petascale threshold (Black, 2025). The capacity was expanded with the Jean Zay H100 supercomputer, upgraded in 2024 to an peak performance of 125.9 petaFLOPS ((CNRS) Cndlrs, 2025). It marks a pivotal milestone in France’s national AI strategy, supporting advanced research in vision, multimodal fusion, and explainable AI across disciplines, while powering key biomedical projects such as large natural language processing, imaging analysis, and protein interaction modeling during the SARS-CoV-2 pandemic (GENCI, 2021). As a publicly funded and domestically operated platform, it reinforces France’s technological sovereignty and leadership in AI innovation.

LUMI is the flagship system of the EuroHPC JU located in Kajaani, Finland, and operating since 2022 (EuroHPC, 2025). With a peak performance of 386 petaFLOPS, it ranked ninth on the TOP500 list as of June 2025 (TOP500, 2025a; EuroHPC, 2025). Powered entirely by hydroelectric energy, it is among the most energy efficient systems worldwide and central to Europe’s green computing agenda (LUMI, 2025). LUMI has played a significant role in onco-pathology research by supporting two large pathology projects, ComPatAI and PanEncoder, which use AI to analyze histopathologic data for early cancer detection, precision diagnosis, and risk stratification (LUMI, 2025; LUMI, 2025). Leonardo is another EuroHPC pre-exascale supercomputer, launched in 2022 in Bologna, Italy, delivering 241 petaFLOPS of peak performance (TOP500, 2025a; Turisini and Cestari, 2023). Through a partnership with Dompe Farmaceutici, Leonardo enables ultra-fast screening of enormous molecular libraries, pandemic response modeling, and rapid drug-target simulations (S.p.A. DF, 2022). Notably, it contributed to Exscalate4CoV project during SARS-CoV-2 pandemic, which advanced an antiviral agent (Raloxifene) into clinical trials (Nicastri et al., 2022).

Launched in 2025 at the University of Bristol, Isambard-AI is the most advanced AI-optimized supercomputer in the UK, delivering over 21 exaFLOPS of AI performance and 216 petaFLOPS for scientific computing (TOP500, 2025a; Caulfield, 2025). It serves as one of the two HPC systems in the AI Research Resource (AIRR) initiative that is funded by the Department for Science, Innovation and Technology, which fosters innovations aligned with national priorities, and is quickly becoming a central resource for life sciences research in the UK (Innovation URa, 2025; McIntosh-Smith and Woods, 2024). Among its early flagship projects, it powers the *Nightingale AI* project at Imperial College, which is developing a sovereign multimodal health foundation model trained on National Health Service data for massive clinical data analysis; the Cancer Screening AI project at University College London, which aims to enhance MRI-based early detection of prostate cancer; and EgoAI at University of Bristol, which leverages AI for wearable device data to assist home care for individuals with cognitive disorders (Caulfield, 2025; Bristol Uo, 2025).

Dawn, commissioned at the University of Cambridge in 2023, is another AI supercomputer that joins Isambard-AI as part of the AIRR, which empowers a wide range of biomedical research, including the development of digital twin models for personalized therapies, treatment response prediction, and large-scale AI workflows for medical imaging and diagnostics, biomarker discovery, and disease stratification (University of Cambridge, 2025a; University of Cambridge, 2025b). Dawn is the chosen platform for one of the UK’s flagship national cancer projects, the UK Cancer Vaccine AI & Supercomputing Project, an ambitious initiative to pioneer the use of AI to create cancer vaccines that are safer, more precise, and more effective (Gudge, 2025).

The most recent and prominent development is the JUPITER system at the Jülich Supercomputing Center in Germany, which is Europe’s first exascale-class system delivering approximately 1 exaFLOPS of peak performance and ranking fourth on the TOP500 List as of November 2025 (TOP500, 2025a; EuroHPC, 2025). Notably, it is recognized as the most energy-efficient supercomputer, powered entirely by renewable energy sources (European Commission, n.d.). JUPITER is leveraged for biomedical research, for example, it enables atomistic simulations of the nuclear pore complex to inform drug development and delivery, powers an AI-driven project of digital biomarker analysis and real-time fatigue monitoring in cancer patients using wearable data, and supports large brain foundation models that link imaging with cellular anatomy to study cognition and neurological disease (Centre JS, 2025a; Centre JS, 2025b). With its ability to train large AI models and run complex multimodal simulations, JUPITER represents a significant step in computational medicine and translational research.

Furthermore, there are more sovereign supercomputers across Europe, collectively driving advances in biomedical research. MareNostrum 5, located in Spain, is a pre-exascale system that actively supports molecular modeling, prediction of genetic variant functions, and molecular-based personalized medicine (BSC, 2025). MeluXina, based in Luxembourg, also contributes to data-driven personalized medicine as well as breast cancer genomics and the discovery of novel antibiotics, positioning itself as a versatile resource for academic and industry-led life science projects (LuxProvide, 2025). Gefion, in Denmark, is an AI-optimized system supporting protein engineering, synthetic biology, and large-scale data analysis aimed at improving women’s health (DCAI, 2025a; DCAI, 2025b). The University of Edinburgh also announced to be the host of UK’s upcoming national supercomputer supported by major government investment (University of Edinburgh, 2025). Collectively, these systems highlight Europe’s strategic alignment toward AI-driven architectures and its leadership in sovereign HPC for biomedical innovation.

#### 3.2.2 Access and governance of HPC in Europe

European governance of HPC is largely anchored by the EuroHPC JU, which oversees the procurement, deployment, and allocation of large computing systems across the EU to strengthen technological sovereignty and innovation (EuroHPC, 2025). The legal foundation was established by Council Regulation (EU) 2018/1488 in 2018 and later amended by Council Regulation (EU) 2024/1732 in June 2024 to expand its mandate to AI infrastructure (Union, 2018; Union, 2024). An important pillar is the European Processor Initiative (EPI), a multi-partner effort to develop low power, high performance, processors and accelerators for exascale and AI workloads, advancing a fully European hardware stack that enhances performance, energy efficiency, and long-term technological sovereignty (Consortium EPI, 2025). Access to EuroHPC supercomputers is merit-based, structured through competitive calls and transparent peer-review process managed by national supercomputing centers along with EuroHPC to ensure fairness and scientific quality (E, 2025). Public sector institutions, eligible enterprises, and industry partners in EU-funded research are qualified for free access to EuroHPC supercomputers, contingent upon adherence to open science principles and public dissemination of results (E, 2025). Council Regulation (EU) 2021/1173 further mandates that all use of EuroHPC resources, including by foreign institutions, must serve European scientific, economic, and strategic interests while preserving autonomy in computing and data capabilities (Union, 2021). This framework reflects the EU’s broader policy commitment to technological sovereignty.

International access to European HPC systems is governed by strict conditions and must occur through formal agreements and full compliance with Union law to protect European interests (Union, 2018). The EuroHPC Access Policy of April 2025 reinforces these provisions by limiting access for institutions outside the Union unless their activities demonstrably benefit Europe and comply with data protection and cybersecurity requirements (E, 2025). These workflows are also governed by broader EU data frameworks, such as the General Data Protection Regulation (GDPR) and European Health Data Space (EHDS) regulations, which mandate stringent safeguards over the handling of personal and health-related data (Schlünder et al., 2025). Together, these instruments ensure that international cooperation remains possible under clearly defined conditions that protect European interests, sensitive data, and support the Union’s broader sovereignty goals. However, persistent challenges remain in harmonizing software licensing, medical data sharing, and cross-border consent mechanisms (Royo et al., 2025). As demand for AI-driven biomedical research intensifies, the creation of flexible and integrated Union-wide governance models will be critical to balance national oversight with scientific agility.

### 3.3 Asia

Asia hosts several sovereign supercomputers that rank among the most powerful and architecturally distinctive systems worldwide. China and Japan lead the region’s computational initiatives, deploying advanced platforms that integrate national strategic goals with applications in biomedical research, epidemiology, genomics, and therapeutic discovery.

China has been a global pioneer in sovereign supercomputing, with national efforts dating back to the 1986 launch of the 863 Program by the Ministry of Science and Technology to strengthen domestic capabilities and reduce reliance on foreign technology (China MoSaTotPsRo, 2025). This strategy yielded major results by 2010, when the Tianhe-1A system at the National Supercomputing Center in Tianjin topped the TOP500 list, signaling China’s emergence as a global leader (TOP500, 2025b). This leadership culminated in 2016 when Chinese systems occupied the top three global rankings and marked a significant milestone for the country’s technological independence (TOP500, 2015). Central to this infrastructure is the Tianhe series, beginning with Tianhe-1A developed by the National University of Defense Technology (NUDT) at the NSC in Tianjin, which features a hybrid CPU-GPU architecture and a proprietary high-speed interconnect developed domestically (Yang et al., 2011). Building on that foundation, Tianhe-2A, upgraded in 2017 at the NSC in Guangzhou, achieved a bigger leap in performance and core density (Russell, 2025). It is leveraged for genomics, molecular dynamics, climate modeling, and drug screening (Liu et al., 2016). Looking ahead, though not publicly benchmarked, China is advancing the development of Tianhe-3, a forthcoming exascale system that can potentially be one of the leading HPCs globally (Mann, 2020; Trader T, 2025).

The Sunway series represents China’s independent trajectory toward exascale computing. Beginning with the 2011 Sunway BlueLight MPP, powered by the country’s first domestically developed ShenWei processor (Min et al., 2013). This effort culminated in Sunway TaihuLight in 2016, which became the world’s fastest supercomputer at the time with a peak performance of 93 petaFLOPS, built entirely with Chinese technologies (Trader T, 2025). TaihuLight contributes significantly to biomedical research, including enhancing quality control for massive sequencing data, deep learning-based histopathology analysis, and molecular simulation for therapeutic discovery (Yan et al., 2025; Li et al., 2021). The most forefront in this roadmap, Sunway OceanLight marks China’s leadership role in the exascale era with domestic architecture, though technical details remain undisclosed. Today, China operates a network of national supercomputing centers (NSC) across major cities, providing a solid foundation for China’s long-term commitment to technological independence and growing leadership in global supercomputing (Liu et al., 2016).

Japan has been a long-standing leader in HPC with development initiatives dating back to the 1980s. A landmark achievement came in 2002 with the deployment of the Earth Simulator, which at the time became the world’s fastest supercomputer (Igarashi, 2025). This leadership position of Japan was reaffirmed with the launch of the K computer in 2011 and, more recently, Fugaku supercomputer, launched in 2020 at the RIKEN Center for Computational Science (Kobayashi et al., 2015). Fugaku achieved a peak performance of 442 petaFLOPS, led the TOP500 rankings from 2020 through 2021, and remains in the top 10 as of 2025 (TOP500, 2025a). It played a critical role during the SARS-CoV-2 pandemic, which supported aerosol dispersion simulations that guided policy-making and anti-viral drug screening; it also powers simulations of brain circuitry to investigate neurological mechanisms (Igarashi, 2025; Rahul and Makoto, 2024).

Other Asian countries have also made significant strides in developing HPC infrastructure. India’s National Supercomputing Mission is building a distributed HPC network to expand indigenous research capacity (National Supercomputing Mission, 2026). Within this ecosystem, the AIRAWAT AI supercomputer is designed for healthcare, while the PARAM series serves as a nationwide backbone for simulations and data intensive research across institutions (National Supercomputing Mission, 2026; Delhi P, 2026). The United Arab Emirates has adopted an aggressive sovereign AI strategy, exemplified by the Falcon family of large language models and the Condor Galaxy supercomputing network, which enables rapid training of large domain specific systems and multilingual models, explicitly positioned to support applications such as genomic analysis and healthcare AI (G42, 2026; Technology Innovation Institute, 2026).

## 4 Future directions

### 4.1 From black box to glass house: building trust in supercomputing

The US expects federally funded projects to uphold principles of transparency, confidentiality, and accountability (Office of Management and Budget, 2025). However, HPCs possess inherent layers of complexity that complicate adherence to traditional data stewardships, such as volatile software stacks, system-specific code environments, and constrained usage time (Barba, 2021). Recognizing these challenges, the Association for Computing Machinery US Public Policy Council (USACM) issued a 2017 statement on algorithmic transparency and accountability, which outlines seven foundational principles: “*Awareness*,” “*Access and redress*,” “*Accountability*,” “*Explanation*,” “*Data provenance*,” “*Auditability*,” and “*Validation and Testing*.” (Association for Computing Machinery US Public Policy Council (USACM), 2025) While not legally binding, it has become widely referenced in policy and ethics discussions on governing large scale data on federal computing platforms. Building on these foundations, many scholars have proposed concrete practices for researchers, such as full documentation and release of code and hardware configurations with persistent identifiers, and timely sharing of outputs through open repositories or preprint platforms to promote peer validation (Barba, 2021). Furthermore, expert and clinical judgment, together with robust validation and cross checking, remain essential to mitigate systematic error at scale and ensure reliable insights from AI-enabled supercomputing.

### 4.2 Fast supercomputing vs. green supercomputing?

HPCs, especially those at the exascale level, present significant environmental challenges due to their immense energy consumption, heat generation, and resulting carbon footprint. These systems consume terawatts of electricity and require extensive cooling infrastructure, contributing to increased greenhouse gas emissions, water use, and pressure on national power grids (Bashroush and Lawrence, 2020; Sun et al., 2024). According to the International Energy Agency (IEA), data center accounted for 1.5% of global electricity consumption, predominantly the US (45%), followed by China and Europe (Agency IE, 2025). Fortunately, global awareness of the potential environmental harms has increased, and large data centers have increasingly implemented measures to address these challenges (TOP500, 2025c). ORNL in the US implemented waste heat recovery by integrating heat pump technology with liquid cooling to repurpose excess heat from Frontier to residential, commercial, or industrial facilities (Sun et al., 2024). Europe consistently prioritizes sustainability in HPC design, exemplified by the Climate Neutral Data Center Pact, a voluntary pledge to achieve climate neutrality by 2030 through power usage efficiency targets and the use of renewable or hourly carbon free energy, with signatories including the flagship system LUMI (Pact CNDC, 2025). German also implements strict rules on energy economization, leading to the deployment of JUPITER, Europe’s first exascale system and the world’s most energy efficient supercomputer, powered entirely by renewable energy (European Commission, n.d.). These initiatives reflect a growing commitment to sustainable supercomputing; while long-term progress requires broad consensus and share responsibility across data centers to balance computational efficiency with environmental impact.

### 4.3 Opening the gates: toward globalized HPC access

Although sovereign supercomputers primarily serve national priorities, efforts have been made to broaden global participation in advanced computing and reduce resource disparities. In Europe, the EuroHPC JU provides free access to supercomputing systems for users from EU Member States and countries associated with the Digital Europe or Horizon Europe Programs, extending eligibility to regions in the Middle East, North Africa, and Asia (E, 2025; European Commission, 2025; Eblen, 2025). In the US, international collaboration is permitted under strict DOE regulations, such as DOE Order 142.3B, which mandates identity verification, sponsor vetting, and security clearance for foreign nationals. DOE Order 486.1A also restricts involvement in foreign government-affiliated programs without exemption (U.S. Department of Energy, 2021; U.S. Department of Energy, 2020). Projects need to be led by a US-based Principal Investigator, with further restrictions that may apply under the Department of Defense regulations (Foundation NS, n.d.). Asia is also actively fostering the global HPC advancement. Japan’s RIKEN Center for Computational Science has partnered with ASEAN member states by providing access to Fugaku and organizing dedicated training for researchers from Southeast Asia (Tan Tin Wee et al., 2021). In parallel, China’s Digital Silk Road, part of the One Belt One Road Initiative, expands digital infrastructure to developing regions through investments in fiber optic networks, cloud platforms, and data centers (Cheng, 2022). These efforts reflect a global strategy that seeks to expand HPC coverage to under resourced communities. However, disparities persist, especially in smaller institutions that often lack technical expertise, experience with access protocols, and sustained resource allocation. To ensure that sovereign HPC access is truly transformative rather than nominal, parallel investment in training, governance, and local capability building is needed, alongside inclusive system design and coordinated international collaboration to support equitable participation in biomedical research.

## 5 Conclusion—roadmap to the future and priority actions

Sovereign supercomputers are becoming central to biomedical innovation, enabling nations to investigate disease mechanisms with unprecedented depth and speed. Nations are launching AI for science strategies on a scale comparable to historic endeavors such as the trilateral collaboration amongst the U.S.–U. K.–Canada for the Manhattan Project, creating infrastructures designed to transform the lives of patients (Goldman Sachs, 2026). This review highlights major HPC systems across North America, Europe, and Asia and their contributions to imaging analysis, molecular simulation, multimodal model development, and precision therapeutic engineering, alongside a clear shift toward AI centric architectures. The growing amalgamation of compute across large data centers is reshaping how computational capacity is delivered. Looking forward, emerging integration with quantum computing is gaining traction, with major pharmaceutical companies and research collaborations exploring hybrid quantum classical approaches through platforms such as International Business Machines (IBM) to enhance molecular simulations. Although still at an early stage, these developments point to a future in which quantum computing augments HPC and has the potential to address currently intractable problems in drug discovery. As computational paradigms evolve, open-source frameworks, such as PyTorch and BioNeMo, remain a critical interface between infrastructure and research. While national strategies establish the infrastructure and priorities, open ecosystems more directly influence research by translating advanced computing into accessible tools and widely adoptable scientific workflows, with their impact is further amplified when combined with HPC resources.

To realize this vision, several priorities will guide policy and investment: First, adoption of concrete technical standards and governance frameworks is required for interoperability across sovereign systems. This includes the implementation of federated data sharing models and containerized, portable workflows (e.g., Docker and Singularity based environments) to enable deployment across heterogeneous computing settings, alongside alignment with established frameworks and standards such as the Global Alliance for Genomics and Health (GA4GH), HL7 Fast Healthcare Interoperability Resources (FHIR), and standardized clinical vocabularies. Second, extending access to researchers in regions without large scale infrastructure will require hybrid models, including cloud federated HPC, subsidized compute access programs, and international training initiatives to build sustainable local expertise. Third, trust must be embedded from the outset through enforceable standards for security, transparency, auditability, and reproducibility, operationalized via regulatory compliance requirements, formal certification and accreditation processes, mandatory audit trails and logging, standardized reporting and documentation protocols, and routine independent oversight and validation. Fourth, results should be shared in a timely manner through standardized mechanisms such as interoperable data repositories and preprint platforms, while cross national validation should be incentivized through coordinated consortia, shared protocols, and requirements for external replication. Fifth, next generation systems must balance performance with energy sustainability to ensure long term viability. Finally, maintaining purpose and focus for key exemplar projects that demonstrate public benefit, such as those to create safer, more precise, and more effective treatments. These efforts will determine whether the extraordinary promise of sovereign AI supercomputers becomes a foundation for global health progress, heralding a new era of biomedical discovery for the benefit of those with diseases such as cancer and yield great public benefit across the world.
